# Demographic, social cognitive and social ecological predictors of intention and participation in screening for colorectal cancer

**DOI:** 10.1186/1471-2458-11-38

**Published:** 2011-01-14

**Authors:** Tess A Gregory, Carlene Wilson, Amy Duncan, Deborah Turnbull, Stephen R Cole, Graeme Young

**Affiliations:** 1 School of Psychology, University of Adelaide, Adelaide, 5000, Australia; 2 Flinders Centre for Cancer Prevention and Control, Flinders University, Bedford Park, 5042, Australia; 3 CSIRO, Preventative Health Research Flagship, Adelaide, 5000, Australia; 4 Bowel Health Service, Repatriation General Hospital, Daw Park, 5041, Australia

## Abstract

**Background:**

Previous research points to differences between predictors of intention to screen for colorectal cancer (CRC) and screening behavior, and suggests social ecological factors may influence screening behavior. The aim of this study was to compare the social cognitive and social ecological predictors of intention to screen with predictors of participation.

**Methods:**

People aged 50 to 74 years recruited from the electoral roll completed a baseline survey (n = 376) and were subsequently invited to complete an immunochemical faecal occult blood test (iFOBT).

**Results:**

Multivariate analyses revealed five predictors of intention to screen and two predictors of participation. Perceived barriers to CRC screening and perceived benefits of CRC screening were the only predictor of both outcomes. There was little support for social ecological factors, but measurement problems may have impacted this finding.

**Conclusions:**

This study has confirmed that the predictors of intention to screen for CRC and screening behaviour, although overlapping, are not the same. Research should focus predominantly on those factors shown to predict participation. Perceptions about the barriers to screening and benefits of screening are key predictors of participation, and provide a focus for intervention programs.

## Background

Colorectal Cancer (CRC) is the most commonly diagnosed internal cancer in Australia affecting both men and women, and the second most common cause of cancer-related death in the Western world [1]. Given the slow progression of the disease, strategies currently focus on the early detection of curable lesions through screening, using endoscopic means or faecal occult blood tests (FOBTs). FOBTs detect minute amounts of blood in the stool; this facilitates the detection of neoplasia at early, curable stages [2]. Population screening with FOBT has been shown to reduce mortality by 15-35% assessed on an intention-to-screen basis relative to an unscreened population [3]. Moreover, the newer immunochemical FOBTs achieve better overall performance than conventional guaiac-based tests [4], making them more effective for use in colorectal cancer screening. Nonetheless, the efficacy of these screening programs depends in part on high participation rates and in many cases, this has not been achieved. For instance, the participation rate in the initial roll-out of the Australian National Bowel Cancer Screening Program (NBCSP) was 40% at 38 weeks post-invitation [5], which is sub-optimal but comparable to national screening programs in other countries [6]. These low participation rates highlight the need to understand the factors that influence the decision to screen for CRC, and ultimately to develop strategies to increase participation rates.

Many studies have established that demographic variables, such as higher income and education, increase the probability of screening for CRC [7]. Understanding demographic associations with screening can be useful in identifying segments of the population to target for intervention programs [8]. On the other hand, psychosocial variables, such as attitudes towards screening and beliefs about the efficacy of screening tests, provide a focus for the development of cognitive, attitudinal and behavioral change programs. A range of behavioral interventions have been found to increase participation in cancer screening programs [9] and growing evidence suggests that such interventions are most effective if based on social cognitive models [10].

Social cognitive models propose that decisions to engage in health promoting behaviours are influenced by psychological factors such as self efficacy, perceived susceptibility to and perceived severity of the disease, and perceived barriers of the preventative health behavior [11]. Research has established that many of these factors influence the decision to screen for CRC [7]. However, social cognitive models have been criticised because variables from these models tend to predict *behavioral intention *better than behavior [11]. A review by Sheeran [12] estimated the average correlation between intention and behavior at 0.53, suggesting that intention only explains about 28% of the variance in behaviour. Moreover, studies have shown that the factors that predict intention to screen are not necessarily the same factors that predict participation in screening programs [13]. These outcomes highlight the need to explore additional predictors of participation in CRC screening. One possibility is to move beyond the intrapersonal level to explore factors associated with participation in screening at a wider, systems level using social ecological models.

Social ecological (SE) models recognise that decisions about engaging in health promoting behaviours, can be influenced at the interpersonal, institutional, community and public policy levels [14]. Several studies have found that subjective norms and social support (*interpersonal level variables*) are associated with CRC screening adherence [15,16]. Institutional level factors, such as endorsement of CRC screening by a primary care physician, have also been shown to influence participation rates [17-19]. These studies support the use of the SE model in understanding intention and behavior around screening for CRC and these factors are particularly important because unlike demographic factors, many are modifiable.

### Aims

Although a substantial amount of research has been conducted to identify variables that predict intention to screen and screening participation, there has been little research that focuses on the difference between behavioral intention to participate and participation. There have been a number of factors identified that predict intention, however, these are not always the same factors that discriminate participants from non participants [13]. Moreover, many studies focus purely on individual level, social cognitive factors, failing to consider more distal influences on the follow through from a decision to screen to actual behaviour. The aims of this study were to (1) examine and compare the demographic and social cognitive predictors of *intention to screen *for CRC and *screening behavior*, and (2) to consider additional predictors operating at a range of levels in the community - such as the social context within which the behavior occurs - based on the Social Ecological Model [14].

## Methods

### Study Population

The Australian Electoral Commission (AEC) provided a list of names and addresses for all people aged 50 to 74 years who were residing in southern urban Adelaide, South Australia (approximately 100,000 records) in early 2006. Since the Australian Federal Government was conducting a pilot National Bowel Cancer Screening Program at the same time, areas outside the Federal screening program were chosen. From the AEC list, a random sample of 1,250 people was selected for the current study. The study population consisted of 602 males (48%) and 648 females, a balance which is representative of those aged 50-74 years in South Australia [20].

### Study Design

This study was approved by ethics committees at the University of Adelaide and the Repatriation General Hospital. All participants were mailed an advance notification letter, followed by a Bowel Cancer Screening Questionnaire (BCSQ), two-weeks later. Non-respondents were followed up with reminder letters six weeks after the mailing of the BCSQ, and with a reminder phone call two weeks after the reminder letter. Participants who returned the survey were then mailed an immunochemical faecal occult blood test (iFOBT also known as a faecal immunochemical test for haemoglobin [FIT], InSure™, Enterix Australia) four weeks after the completed BCSQ had been received. People who did not return FIT samples after six weeks were sent a reminder letter.

### Materials

#### The Bowel Cancer Screening Questionnaire (BCSQ)

The BCSQ was designed to determine a participant's intention to screen for CRC and collect information on a wide range of demographic, social cognitive and social ecological measures that have been shown to be associated with intention to screen or screening participation in previous research. The BCSQ builds on previous work by our research group [21] and was developed after extensive consultation with health professionals and piloting. The full BCSQ is available from the corresponding author.

Intention to screen was measured by stage of readiness to screen for CRC based on the Trans-Theoretical Model of behavior change [TTM; [22]]. Multi-stage models such as the TTM assume that a decision to engage in a health-related behavior involves several discrete stages and thus the TTM provides a comprehensive measure of behavioral intention. The terms intention and stage of change will be used interchangeably, hereinafter. Five forced-choice questions (e.g. "Have you ever thought about screening for colorectal cancer?", "If you have thought about screening for colorectal cancer, have you made a decision?") were used to classify participants into one of six stages of readiness to screen. The six stages were (1) *Pre-contemplation; *have not considered screening for CRC, (2) *Contemplation*; have thought about screening for CRC but have not made a decision, (3) *Preparation*; have decided to screen with FOBT, (4) *Action*; have already screened for CRC using an FOBT, (5) *Rejection*; have thought about screening for CRC but have decided not to, and (6) *Colonoscopy intention*; have thought about screening with FOBT but have decided to complete a colonoscopy instead.

The BCSQ included eight demographic questions (age, gender, marital status, employment status, education, birth country, language spoken at home, and private health insurance status) and two questions concerning previous experience with cancer: (1) have you had any cancer screening tests in the past (yes/no), and, (2) have you known someone who has had bowel cancer (yes/no)? In addition, postcodes for all potential invitees were provided by the AEC and were converted to Socio-Economic Indexes for Areas (SEIFA) codes, to provide a measure of socio-economic status (SES). The SEIFA Index of Relative Socio-economic Disadvantage and Advantage classifies postcodes into deciles from 1 (most disadvantaged) to 10 (most advantaged) based on household income, education, unemployment and unskilled occupations [23]. SES was split into three groups based on the deciles (low SES = 1 - 3, average SES = 4 - 6, and high SES= 7 to 10) for the statistical analyses.

A social cognition scale was created to measure a range of social and cognitive factors purported to influence intention to screen for CRC and/or screening behaviour (31-items). A social ecological scale was created to measure the more distal influences on intention and screening behaviour (29-items). All items were measured on 5-point likert scales ranging from 'strongly disagree' to 'strongly agree'.

### Screening Offer

The screening package included (a) a bowel cancer screening information pamphlet, (b) an InSure™ FIT, (c) a combined Participant Details and Consent Form confirming personal details, nominating a preferred doctor for follow up and consent to obtain clinical follow-up reports if required, (d) a reply-paid return envelope. The Bowel Health Service (BHS) informed the participant if the result was negative, and both the participant and their preferred doctor if FIT positive, and assisted with arranging clinical follow-up. Participation was defined as a returned and completed FIT within 12 weeks of the original offer.

### Statistical Analyses

To reduce the social cognitive and social ecological items to a smaller number of factors, exploratory factor analyses were undertaken using PASW Statistics v18. Factor scores were generated using the regression method in PASW. Following this, demographic variables were analysed for their univariate association with intention to screen and participation using χ^2 ^tests. Latent factors were analysed for their univariate associations with intention to screen and participation using one-way ANOVAs and independent samples t-tests. All significant predictors from the univariate models were included in multivariate generalised linear models to determine joint predictors of intention to screen and participation. For *intention*, pre-contemplators were compared to people in the two higher stages of readiness to screen. For *participation*, non-participants were compared with participants to examine the relative risk of predictors on participation.

## Results

### Response rates

**Figure 1:**
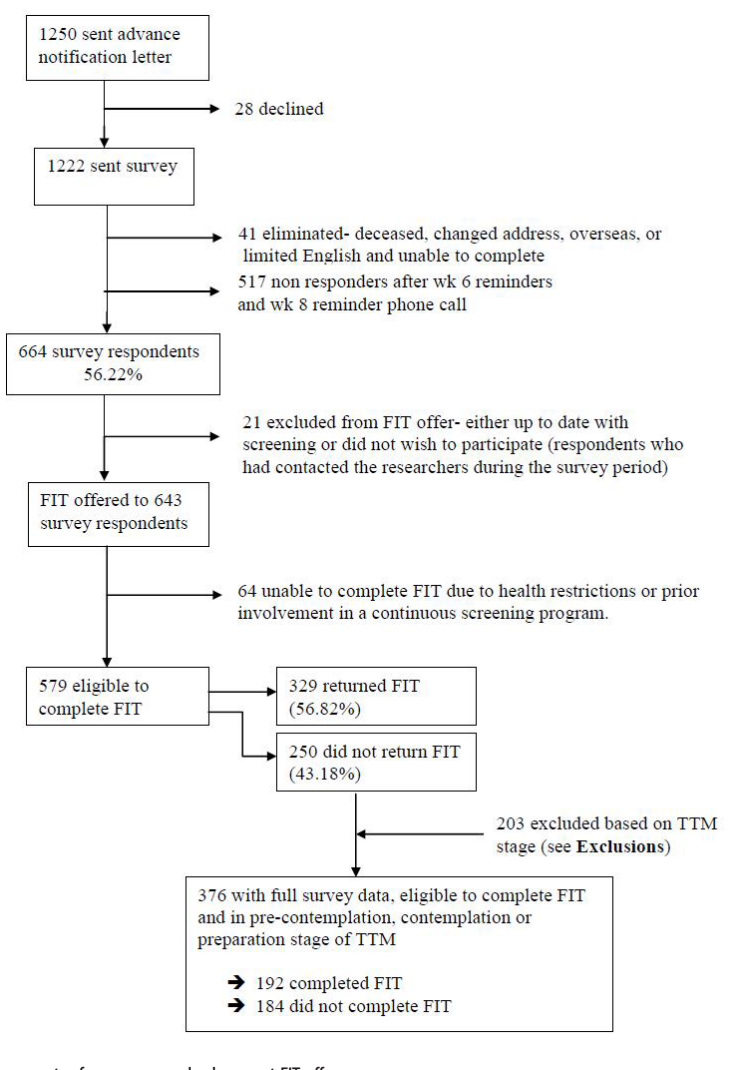
**Response rates from survey and subsequent FIT offer**.

Figure 1 shows the response rate from the questionnaire and subsequent FIT offer. Surveys were received from 664 participants giving an adjusted response rate of 56% (664/1,181). Twenty-one people were excluded from the FIT offer, and 64 people were subsequently unable to complete the FIT (see Figure 1), leaving 579 eligible to complete the screening test. After 12-weeks, 329 participants had returned a completed FIT giving an adjusted participation rate of 57% (329/579).

### Exclusions

Participants were classified into one of six groups to measure stage of readiness to screen (precontemplation, contemplation, preparation, action, rejection and colonoscopy intention). Given that we were interested in a relatively naive sample, people who were in the *action*, *rejection *or *colonoscopy intention *stages were excluded from all further analyses as they had reached a higher decision stage. After exclusions, we had a final sample of 376 people with full survey data, who were eligible to complete the FIT and who were classified into precontemplation (n = 215), contemplation (n = 110) or preparation (n = 51). Of these participants, 192 completed the FIT (51%) and 184 did not.

### Description of the sample

The final sample (n = 376) contained approximately equal numbers of males (48%) and females (52%). Just over half of the participants (58%) were aged in their 50s, about one third (32%) were aged in their 60s and 10% were aged over 70 years. The majority were born in Australia (73%), spoke English at home (90%), and were in a married or defacto relationship (75%). About 58% of participants were still in paid employment (39% full-time) and 35% were retired or semi-retired. Almost all participants had completed high school (93%), and about half the participants had completed further education (26% technical certificate/diploma, 19% tertiary education). The sample was somewhat over-represented by people in the most advantaged suburbs (51% in high SES group) and under-represented by people in the most disadvantaged suburbs (12% in low SES group.

### Identification of factors

#### Social cognitive factors

The 31-items from the social cognitive scale were suitable for factor analysis, according to the KMO (.718) and Bartlett's Test of Sphericity (χ^2 ^(465) = 2549.5, *p *< .001). Examination of the scree plot of eigenvalues suggested a six factor solution with eigenvalues of 4.18, 2.72, 2.12, 1.89, 1.62, and 1.50. We extracted a six factor solution and used a varimax rotation. All factors were defined by at least 3 items. Only one item did not load significantly on any of the six factors. We examined 7, 8 and 9-factor solutions because there were 9 eigenvalues above 1.0. However, these solutions resulted in items loading weakly across several factors. The six-factor solution was clearly the most interpretable. Factor scores were created using the regression method is PASW.

Table 1 shows the factor loadings and questionnaire items for the social cognitive factors. Factor 1 was labelled *barriers/benefits *and was defined by positive agreement with barriers to CRC screening (i.e. embarrassing, time consuming, inconvenient, fear) and negative agreement with the benefits of CRC screening (i.e. reassuring, convenient). Factors 2, 3 and 4 were labelled *chance health locus of control (HLC)*, *powerful others HLC *and *internal HLC*. Factor 5 was labelled *perceived susceptibility *and could be thought of as similar to risk perception in other models. Factor 6 was labelled *CRC knowledge *and was defined by items about knowledge of CRC and CRC screening.

**Table 1:** Item loadings for the factor analysis and questionnaire items (Social Cognitive scale)

Factor and loading						Questionnaire items
	
F1	F2	F3	F4	F5	F6	
-.50						I feel confidence that I wouldn't find the test overly distasteful or embarrassing
.56						I think that giving a sample of faeces to another person for bowel cancer screening is embarrassing
-.69						I feel confident that I would be able to find time in the day to complete the test
-.56						Screening for bowel cancer can pick it up early when cancer can be treated
-.55						I think that having a faecal occult blood test would be reassuring
.53						I believe that testing faeces for the purpose of bowel cancer screening is unhygienic
-.57						It is quite convenient that I can screen myself for bowel cancer at home
.47						Fear of cancer would put me off having a faecal occult blood test
.69						I think that faecal occult blood tests are inconvenient
.47						Screening for bowel cancer is time consuming
	.42					There are things I care about more than my health
	.43					My family has a lot to do with my becoming sick or staying healthy
	.54	.41				No matter what I do, if I'm going to get sick, I will get sick
	.62	.31				My good health is largely a matter of good fortune
	.46	.35				When I am sick, I just have to let nature run its course
		.60				When I recover from an illness, it's usually because other people have been taking good care of me
		.67				Having regular contact with my doctor is the best way for me to avoid illness
		.68				Regarding my health, I can only do what my doctor tells me to do
		.39	.44			If you don't have your health, you don't have anything
			.41			When I get sick, I am to blame
			.57			I am in control of my health
			.66			If I become sick, I have the power to make myself well again
			.66			If I take the right actions, I can stay healthy
				.63		Most things that affect my health happen to me by accident
				.76		There is a good chance that I will get bowel cancer
				.76		I worry a lot about getting bowel cancer
				.81		My chance of getting bowel cancer is high
				.33	-.34	People who get bowel cancer usually die soon
					.82	I believe I know a lot about bowel cancer
					.83	I know a lot about screening for bowel cancer

Note. F1 = *Barriers/Benefits*, F2 = *Chance Health Locus of Control (HLC)*, F3 = *Powerful Others HLC*, F4 = *Internal HLC*, F5 = *Perceived Susceptibility*, F6 = *CRC Knowledge*

#### Social ecological factors

The 29 social ecological items were also suitable for factor analysis, with a KMO of .829 and a significant Bartlett's Test of Sphericity (χ^2 ^(406) = 3163.1, *p *< .001). The scree plot suggested a three factor solution with eigenvalues of 6.06, 2.83 and 1.65. We extracted a three factor solution and applied a varimax rotation. All factors were defined by at least seven items suggesting that we may have extracted too few factors. Therefore, we examined 4 and 5 factor solutions but these reduced the interpretability of the solution so we selected the 3 factor solution.

Table 2 shows the factor loadings and questionnaire items for the social ecological factors. Factor 1 was labelled *social support *and indicated support from family and friends. Factor 2 was labelled *barriers to accessing GPs *including financial, cultural and language barriers to screening.

**Table 2:** Item loadings for the factor analysis and questionnaire items (Social Ecological Scale)

Factor and loadings			Questionnaire items
	
F1	F2	F3	
.39		.54	I feel comfortable talking to my GP about my own health
.79			I get the emotional help and support I need from my family
.69			I can count on my friends when things go wrong
.84			I can talk about my problems with my family
.83			My family is willing to help me make decisions
.66			I can talk about my problems with my friends
.46			There is a special person with whom I can share my joys and sorrows
.65	-.32		My family will support me if I decide to screen for bowel cancer
.46	-.30		People from my background would support my decision to screen for bowel cancer
	.60		I am unable to see a GP every time I need to because I am disabled and there is no one to take me there
	.33		My family thinks people with my background should not screen themselves for bowel cancer
	.43		I have a chronic disease so don't think about bowel cancer
	.36		People from my background think you can't do anything about cancer
	.73		It is difficult for me to visit my GP because I do not speak English very well
	.67		My GP lives very far from me so I cannot see him as often as I need
	.73		In my religion, cancer is a punishment and I cannot do anything about this
	.61		I think it is suspicious that the government supports screening for bowel cancer
	.51		It is difficult to get to the hospital from my home
	-.32		I can get to the pharmacy easily from my home
	.42	-.38	It is difficult for me to see my GP regularly because I do not have a car
		-.50	I am unable to see a GP every time need to because it is too expensive
		.55	I generally trust doctors
		-.63	I do not often visit doctors because they make me anxious
		.49	I have access to the health care provider who can answer the questions I have about bowel cancer
		-.62	It is difficult for me to find time to see a GP

Note. F1 = *Social support*, F2 = *Barriers to accessing GPs*, F3 = *Trust in GPs*

Factor 3 was labelled *trust in GPs *with high scores representing trust and being comfortable with one's GP.

### Univariate predictors of intention and screening behavior

#### Demographic and background variables

The impact of demographic and background variables on intention to screen and screening behaviors was measured using χ^2 ^analyses (see Table 3). Intention to screen was significantly associated with five demographic variables: gender, country of birth, language spoken at home, past screening for cancer and knowing someone with bowel cancer. Participation in the screening offer was associated with just two demographic variables: past screening for cancer and knowing someone with bowel cancer.

**Table 3:** Demographic associations with stage of readiness to screen and screening behavior

			Stage of Readiness to Screen							Screening behavior			
		
		Precontemplation (n = 215)		Contemplation (n = 110)		Preparation (n = 51)		χ2	Non-participants (n = 184)		Participants (n = 192)		χ2
		
		n	%	n	%	n	%		n	%	n	%	
**Age**	50 - 54	65	30.2	31	28.4	12	23.5	5.93	62	33.9	46	24.0	8.27
	55 - 59	60	27.9	33	30.3	17	33.3		54	29.5	56	29.2	
	60 - 64	40	18.6	28	25.7	9	17.6		38	20.8	39	20.3	
	65 - 69	25	11.6	10	9.2	7	13.7		15	8.2	27	14.1	
	70 - 75	25	11.6	7	6.4	6	11.8		14	7.7	24	12.5	

**Gender**	Male	106	49.3	43	39.4	32	62.7	7.77*	95	51.9	86	44.8	1.90
	Female	109	50.7	66	60.6	19	37.3		88	48.1	106	55.2	

**Marital**	Married/de-facto	156	73.2	81	75.7	41	80.4	1.17	135	73.8	143	76.1	0.26
**status**	Non-married^1^	57	26.8	26	24.3	10	19.6		48	26.2	45	23.9	

**Employment**	Full-time	79	36.7	43	40.2	23	45.1	3.33	82	44.8	63	33.2	6.12
**status**	Part-time	42	19.5	19	17.8	9	17.6		34	18.6	36	18.9	
	Home duties/unemployed	18	8.4	8	7.5	1	2.0		12	6.6	15	7.9	
	Retired/Semi-retired	76	35.3	37	34.6	18	35.3		55	30.1	76	40.0	

**Education**	Primary school	17	7.9	9	8.3	1	2.0	5.92	12	6.6	15	7.9	3.55
	High school	101	47.2	49	45.0	3	52.0		81	44.5	95	49.7	
	Technical certificate	54	25.2	28	25.7	16	32.0		48	26.4	50	26.2	
	Uni degree	23	10.7	16	14.7	5	10.0		27	14.8	17	8.9	
	Post-graduate degree	19	8.9	7	6.4	2	4.0		14	7.7	14	7.3	

**Born in**	Yes	147	68.4	94	86.2	34	66.7	13.15**	135	73.8	140	72.9	0.04
**Australia**	No	68	31.6	15	13.8	17	33.3		48	26.2	52	27.1	

**Language**	English	186	86.9	104	95.4	46	93.9	6.78*	163	90.6	173	90.1	0.02
**spoken**	Non-English	28	13.1	5	4.6	3	6.1		17	9.4	19	9.9	

**Private health**	Yes	144	67.6	86	79.6	39	76.5	5.69	131	72.8	138	71.9	0.04
**insurance**	No	69	32.4	22	20.4	12	23.5		49	27.2	54	28.1	

**Socio-Economic**	Low	29	13.6	9	8.2	6	11.8	2.16	21	11.5	23	12.0	4.81
**Status**	Medium	78	36.6	43	39.1	18	35.3		58	31.9	81	42.2	
	High	106	49.8	58	52.7	27	52.9		103	56.6	88	45.8	

**Past cancer**	Yes	115	53.7	79	72.5	34	68.0	11.82**	100	54.6	128	67.4	6.35*
**screening**	No	99	46.3	30	27.5	16	32.0		83	45.4	62	32.6	

**Known someone with**	Yes	117	54.9	79	72.5	37	74.0	12.68**	100	55.2	133	69.6	8.22**
**bowel cancer**	No	96	45.1	30	27.5	13	26.0		81	44.8	58	30.4	

*Note*. Widowed, single, divorced and separated groups were combined into a non-married group due to low cell counts

* p < 0.5, ** p < .01

#### Social cognitive and social ecological factors

Table 4 presents results of the univariate analyses for social cognitive and social ecological factors. Intention to screen was associated with barriers/benefits of screening, chance health locus of control, powerful others health locus of control, perceived susceptibility, CRC knowledge and trust in GPs. All effects were in the expected direction. Specifically, people in the pre-contemplation stage had higher barriers, higher chance health locus of control, lower powerful others health locus of control, lower perceived susceptibility, lower CRC knowledge and lower trust in GPs than people in higher stages of readiness to screen. As with the demographic variables there were fewer significant associations with participation in the screening offer than with intention. Participation was associated with barriers/benefits so people who participated perceived significantly lower barriers to screening and higher benefits to screening than people who did not participate in screening.

**Table 4:** Social cognitive and social ecological associations with stage of readiness to screen and screening behavior

	Stage of readiness to screen		Participation	
	
	F-value	p-value	t-value	p-value
**Social Cognitive**				
Barriers/Benefits	14.73	.01	3.59	.01
Chance HLC	6.81	.01	0.87	.37
Powerful Others HLC	3.55	.03	0.97	.33
Internal HLC	0.15	.86	1.13	.26
Perceived Susceptibility	14.14	.01	1.81	.07
CRC Knowledge	5.18	.01	0.51	.61
**Social Ecological**				
Social Support	0.04	.96	1.25	.21
Barriers to accessing GPs	1.87	.16	1.06	.29
Trust in GPs	5.91	.01	1.24	.22

Note. HLC = Health Locus of Control, GP = General practitioner/physician

### Multivariate models

Table 5 presents results of the final multivariate models predicting intention to screen and screening behavior. There were five significant predictors of stage of readiness to screen for CRC. Specifically, people who (1) had screened for cancer in the past, (2) perceived low barriers and high benefits to screening, (3) believed that good health was not due of chance, (4) people who perceive themselves as susceptible to CRC and (5) had higher perceived knowledge about CRC and screening, were more likely to be in a higher stage of readiness to screen. There were only two significant predictors of participation; people who (1) had known someone with CRC, and (2) perceived low barriers and high benefits to screening were more likely to participate in the screening offer.

**Table 5:** Multivariate predictors of stage of readiness to screen and screening behaviour

	Higher stage of readiness to Screen			Participation in screening offer		
	
	Risk Ratio	p-value	95% CI	Risk Ratio	p-value	95% CI
**Background variables**						
Gender						
Female^a^	1.00	-	-	-	-	-
Male	1.08	.51	0.85, 1.38	-	-	-

Born in Australia						
Yes^*a*^	1.00	-	-	-	-	-
No	0.81	.16	0.60, 1.09	-	-	-

Language spoken at home						
English^a^	1.00			-	-	-
Non-English	0.79	.46	0.42, 1.49	-	-	-

Screened for cancer in past						
No^*a*^	1.00			1.00	-	-
Yes	1.38	.02	1.04, 1.81	1.21	.08	0.98, 1.50

Known someone with CRC						
No^*a*^	1.00			1.00	-	-
Yes	1.23	.15	0.93, 1.63	1.26	.04	1.02, 1.57

**Social Cognitive**						
Barriers/Benefits	0.78	.01	0.70, 0.94	0.86	.01	0.79, 0.95
Chance HLC	0.84	.01	0.75, 0.94	-	-	-
Powerful Others HLC	1.02	.78	0.91, 1.13	-	-	-
Perceived Susceptibility	1.28	.01	1.15, 1.42	-	-	-
CRC Knowledge	1.11	.05	1.01, 1.24	-	-	-
**Social Ecological**						
Trust in GPs	1.08	.23	0.95, 1.22	-	-	-

*Note*. Variables without statistics presented were not included in the final multivariate model

^*a *^Reference group for categorical predictors

## Discussion

This study set out to examine and compare the demographic, social cognitive and social ecological predictors of *intention to screen *for colorectal cancer (CRC) and *screening behavior*. Multivariate analyses revealed five predictors of intention to screen and two predictors of participation. These predictors consisted of demographic and experiential measures and social cognitive variables. None of the social ecological factors was significant multivariate predictors of intention or behavior. Whilst there was some overlap between predictors, our findings support the proposition that predictors of intention to screen for CRC and actual CRC screening behavior are not the same [13]. This suggests that the decision-making process for CRC screening is complex, as the factors that move people from pre-contemplation to subsequent higher stages of readiness to screen do not necessarily move them to engage in health promoting behaviors. Moreover, it suggests that interventions should focus on those factors that predict participation, rather than intention, notwithstanding the possibility that intention is a necessary but not sufficient prerequisite for behavior. These findings may also have implications for CRC screening using colonoscopy, which is recommended in countries such as the US and Germany. Although, the specific factors that predict screening with colonoscopy may be different to those that predict screening with FOBT, the general finding that different factors predict intention and behaviour probably also applies to CRC screening with colonoscopy.

People who had completed different forms of cancer screening in the past were more likely to intend to screen for CRC, and were also more likely to complete the FOBT, although this was only significant in the univariate analysis. Thus involvement in cancer screening programs increases the probability of an individual completing subsequent tests, which is important as screening for most cancers requires regular annual or biennial completion. These results are consistent with a review article by Vernon [7], which reported that a significant percentage of people (above 75% in almost all studies) who completed the first FOBT, completed subsequent tests. It also implies that this health-promoting behavior can transfer from one type of cancer to another. That is, people who had completed screening for any cancer were more likely to screen for CRC suggesting the possible generalisation of self-efficacy, or some other social cognitive predictor. Thus, interventions to increase participation in screening for one cancer may also increase the uptake of CRC screening, and vice versa, increasing the impact of specific intervention programs on population health outcomes.

Despite the criticism often levelled at social cognitive models [11], *perceived barriers to and benefits of screening *predicted participation in this sample. People who did not perceive large barriers to screening and believed in the benefits of screening were more likely to screen. Both perceived benefits and barriers are theoretically amenable to change, and thus intervention programs may benefit from educating the public about the benefits of screening for CRC, while attempting to allay their concerns about the barriers to CRC screening. Intervention programs that successfully modify perceptions about benefits of and barriers to CRC screening should lead to an increase in the participation rates for CRC screening.

Social ecological models aid in moving beyond the individual level, to allow a focus on factors that influence health behaviors at the interpersonal and community levels [24]. This study evaluated the impact of several social ecological factors (social support, barriers to accessing GPs and trust in GPs) on intention to screen and screening behavior. Contrary to findings from at least two other studies, social support was not associated with intention or behavior in this sample [15,16]. However, the effect of social support was weak in both, and other studies have found social support has no effect on CRC adherence [25], so this issue remains unresolved. Barriers to accessing GPs were not related to intention to screen or participation in screening, contrary to expectation. This factor was made up of a range of different barriers including financial, cultural, language, disability and time barriers, with most individual barriers measured by just one item. Although all items loaded together in the factor analysis, it may have been better to have multiple items for each type of barrier (financial, language etc) to better define barriers to accessing healthcare in the community and further explore the contribution of social ecological factors to CRC screening behavior.

One final point concerns the representativeness of our sample. Because we were interested in using demographic, psychosocial and social ecological variables as predictors of intention and participation, it was necessary to limit the study sample to those who completed the survey and therefore the predictor variables. It is therefore possible that the sample in this study were qualitatively different to participants in a "real world" national screening program, who are offered screening without completing a survey, and this may have influenced our results. In an earlier analysis of the current sample, Duncan et al. [8] established that gender distribution and SES were comparable in the group who did and did not complete the survey. While we acknowledge that the two groups may have differed on other variables it is difficult, if not impossible, to measure the extent of any potential bias without having detailed information on people who do not complete the survey, which is inherently unavailable.

## Conclusions

This study has confirmed that the predictors of intention to screen for CRC and screening behaviour, although overlapping, are not the same. Although social cognitive factors are strongly related to intention to screen, their link with participation is weak. Beliefs about the benefits of screening and the barriers to completing CRC screening are key predictors of participation, and provide a focus for intervention programs. Institutional and community factors from the social ecological model had limited influence on CRC screening, but improvements in measuring these constructs is needed before any substantive conclusions about their impact can be drawn.

## Competing interests

The authors declare that they have no competing interests.

## Authors' contributions

TG drafted the manuscript and performed the statistical analyses, AD participated in the coordination of the study, preliminary analyses and drafting the manuscript, CW, DT, SC and GY participated in the study design, defining the goals of the study, developing the questionnaire, overseeing the analyses and drafting the manuscript. All authors have reviewed the final submission and are in agreement about the content of the manuscript.

All authors have reviewed the final submission and are in agreement about the content of the manuscript.

## Pre-publication history

The pre-publication history for this paper can be accessed here:

http://www.biomedcentral.com/1471-2458/11/38/prepub
